# Dynamic physiological response of Mongolian pine ectomycorrhizal seedlings to drought and re-watering

**DOI:** 10.3389/fpls.2026.1744853

**Published:** 2026-02-18

**Authors:** Yue Ren, Guang Yang, Guang-lei Gao, Guo-dong Ding

**Affiliations:** 1 College of Desert Control Science and Engineering, Inner Mongolia Agricultural University, Hohhot, Inner Mongolia, China; 2 School of Soil and Water Conservation, Beijing Forestry University, Beijing, China; 3 State Key Laboratory of Efficient Production of Forest Resources, Beijing Forestry University, Beijing, China; 4 Engineering Research Center of Forestry Ecological Engineering, Ministry of Education, Beijing Forestry University, Beijing, China; 5 Key Laboratory of State Forestry and Grassland Administration on Soil and Water Conservation, Beijing Forestry University, Beijing, China

**Keywords:** drought, ectomycorrhizal fungi, Mongolian pine, physiological characteristics, re-watering

## Abstract

Mongolian pine (*Pinus sylvestris* var. *mongholica*) is a key species for ecological restoration in northern China, frequently exposed to cyclical drought-rewatering stress in arid and semi-arid regions. While ectomycorrhizal fungi (EMF) are known to enhance plant drought tolerance, their mechanistic role in mediating Mongolian pine’s response to drought-rewatering cycles remains unclear. In this study, two-year-old Mongolian pine seedlings inoculated with *Rhizopogon* sp. (Rh) and *Tomentella* sp. (To) fungi. Outdoor pot experiments were conducted under controlled soil moisture regimes, establishing five hydrological gradients: well-watered control (CK0), mild drought (D7), moderate drought (D14), severe drought (D21), and extreme drought (D35), following by rewatering 1 day after the end of each stress period. Additionally, we implemented four inoculation treatments: a non-inoculated control, Rh inoculation, To inoculation, and Rh+To co-inoculation. We measured seedling growth, photosynthesis parameters, water, osmotic regulatory substances, antioxidant enzyme activities, as well as drought resistance, drought recovery ability and drought adaptation ability, along with their intercorrelations. The results demonstrated that (1) drought stress significantly reduced seedling photosynthetic, fluorescence parameters, and water potential indicators (*P* < 0.05). Specifically, photosynthetic and fluorescence parameters, leaf relative water content (RWC), and plant water potential declined progressively with increasing stress intensity increased. (2) After re-watering, physiological indicators under different drought stress degrees exhibited varying degrees of recovery. Photosynthetic and fluorescence parameters exceeded those observed during drought stress treatments, while water use efficiency (WUE) and root water potential (ψ_r_) showed complex recovery patterns, in some cases exceeding levels observed in the well-watered control group (*P* < 0.05). (3) EMF inoculation, especially Rh+To, effectively alleviated drought-induced physiological inhibition, enhancing seedling drought resistance, recovery, and adaptation. (4) Drought recovery capability was closely linked to drought adaptation was critical for overall resistance. Maintaining relatively high RWC during drought periods and preserving elevated the maximal photochemical efficiency of photosystem II (Fv/Fm) values during re-watering emerged as key factors for enhancing drought resistance in Mongolian pine seedlings.

## 1 Introduction

In the context of increasing global climate change and ecosystem disruption, drought has become one of the most common and damaging abiotic stressors impacting plant growth, significantly disrupting their normal life processes and metabolic functions (Bogati and Walczak, 2022; Zia et al., 2021). The combination of low rainfall and extreme fluctuations in precipitation makes drought stress a continual challenge in arid and semi-arid areas (Ploughe et al., 2019). In natural settings, the increasing frequency of “drought-to-waterlogging transitions” driven by periodic droughts and sudden heavy rainfall events exposes plants to more complex and extreme fluctuations in water availability (Jia et al., 2024). Consequently, maintaining internal water balance and physiological homeostasis under such dynamic hydrological conditions is crucial for plant survival and healthy growth.

In response to drought stress, plant engage in a range of coordinated physiological reactions, molecular changes, and defense strategies. These strategies enable them to maintain growth, development, and homeostasis through morphological modifications as well as alterations in biochemical and metabolic processes, ultimately enhancing their overall fitness under water-deficient conditions (Agurla et al., 2018; Qi et al., 2021; Mittler et al., 2022). Plant responses to water scarcity occur through coordinated physiological regulation across multiple scales, including osmotic adjustment and stomatal control to photosynthetic efficiency and metabolic homeostasis, which safeguard the plant during drought conditions (Lu et al., 2019; Xiao et al., 2020; Gupta et al., 2020). Plant adaptation to drought environments is reflected not only in resilience during the stress period but also, and more critically, in the capacity for recovery once the stress is alleviated (Wang et al., 2022; Anjum et al., 2017). Drought tolerance is a vital trait that allows plants to survive in conditions with limited water (Liu et al., 2023). The post-drought recovery capacity of plants, manifested through rapid damage repair and physiological compensation during rewatering, represents a crucial component of their drought adaptation strategy in water-limited environments (Sun et al., 2016). After rewatering, plants swiftly restore photosynthesis and growth through various mechanisms such as turnover of cellular components, reopening of stomata, and scavenging of peroxides (Abid et al., 2018; Ruehr et al., 2019; Jia et al., 2021). The recovery capacity is influenced by the intensity and duration of the drought, as well as species-specific adaptive traits (Wang et al., 2025). Consequently, systematically elucidating the mechanisms underlying plant responses and recovery during drought-rewatering cycles is of significant scientific importance for enhancing plant adaptive capacity to climate change and safeguarding ecological security in arid regions.

Mongolian pine (*Pinus sylvestris* var. *mongolica*) is known for its exceptional adaptability and resilience to stress, making it a crucial species for building ecological barriers and restoring degraded ecosystems in the wind-sand region of northern China (Ren et al., 2023). Simultaneously, as a conifer that relies on ectomycorrhizal fungi (EMF), its survival and performance are significantly influenced by these essential fungal partners (Zhao et al., 2022). Mycorrhizal fungi are vital to terrestrial ecosystems, playing a key role in important ecological processes such as biogeochemical cycling and energy flow, while also enhancing ecosystem stability and biodiversity (de Mesquita et al., 2023). It has been demonstrated that EMF can effectively improve the ability of host plants to withstand drought stress and play an irreplaceable role in the process of forest and vegetation restoration and reconstruction in arid and semi-arid regions of China (Tedersoo et al., 2020; Castaño et al., 2023). The mechanisms by which EMF confer drought resilience are multifaceted and may involve: (1) morphological and hydraulic improvements, such as modifying root system architecture to enhance water foraging and uptake efficiency, thereby helping to maintain higher plant water status (Chen et al., 2025; Liu et al., 2021); (2) physiological and biochemical enhancements, including the modulation of stomatal behavior, accumulation of osmolytes for osmotic adjustment, and bolstering of the antioxidant defense system to mitigate oxidative damage (Madouh and Quoreshi, 2023); and (3) potential molecular-level interactions, such as influencing phytohormone signaling pathways (e.g., ABA) or priming the expression of host stress-responsive genes (Fang and Xiong, 2015). Additionally, EMF contribute to seedling recovery after post-drought drought rewatering, improving survival rate (Yin et al., 2018). Therefore, elucidating the specific mechanisms by which EMF strengthen drought stress resistance and recovery after rewatering is essential for enhancing the drought tolerance of Mongolian pine seedlings.

Mongolian pine plantations consistently face challenges related to water stress challenges, which greatly limits their physiological processes and hinders population regeneration (Deng et al., 2021). EMF play a crucial role in how plants respond to drought stress and subsequent rewatering. In light of this, our study focused on Mongolian pine seedlings that were inoculated with drought-resistant fungi. Through controlled outdoor pot experiments with regulated soil moisture levels, our study aims to examine the physiological responses of ectomycorrhizal seedlings to different lengths of drought stress, and evaluate their recovery ability after rewatering. Accordingly, we propose the following hypotheses: (1) Drought stress will lead to a progressive decline in photosynthetic performance, chlorophyll fluorescence, and water status in Mongolian pine seedlings, with the severity of inhibition increasing alongside both the intensity and duration of the stress. (2) Inoculation with EMF-particularly dual inoculation-will significantly mitigate the drought-induced physiological impairments outlined in hypothesis 1. (3) Following rewatering, EMF-inoculated seedlings will exhibit faster and more complete recovery of physiological functions compared to non-inoculated counterparts; furthermore, the degree of recovery will be influenced by the intensity of prior drought stress. We expect our results to shed light on the mechanistic basis of drought resistance, recovery capacity, and adaptation strategies of Mongolian pine ectomycorrhizal seedlings, ultimately providing a deeper scientific understanding and practical approaches for improving the climate resilience and sustainable management of Mongolian pine plantations amid global climate change.

## 2 Materials and experimental methods

### 2.1 Experimental materials

The experimental materials included two-year-old Mongolian pine seedlings procured from Jiahui Nursery (Zhanggutai Town, Liaoning Province, China). The growth substrate was collected from the 0–60 cm soil layer in the undisturbed understory of a Mongolian pine plantation in the same area. The soil was carefully cleared of large debris (rocks, visible roots) and sieved through a 2-mm mesh to ensure homogeneity. It was then sterilized by autoclaving at 121 °C for 30 min to remove resident microorganisms. The physicochemical properties of the soil were as follows: soil pH of 6.85, organic matter content of 7.59 g/kg, total nitrogen content of 0.52 g/kg, total phosphorus content of 0.47 g/kg, ammonium nitrogen content of 1.95 mg/kg, and available phosphorus content of 0.98 mg/kg. The EMF strains *Rhizopogon* sp. (Rh, ATCC 46218) and *Tomentella* sp. (To, ATCC 90471), obtained from the China Agricultural Microbial Culture Collection Center (ACCC), were selected as inoculants for this study based on their ecological and functional significance. Both are commonly associated with conifer roots in northern forests and are known to contribute to nutrient mobilization and host stress tolerance (Tedersoo et al., 2020; Yin et al., 2018). Before inoculation, the freeze-dried fungal cultures were transferred aseptically onto potato dextrose (PD) agar medium in a laminar flow hood. The cultures were then sealed with parafilm and incubated in the dark at 25 °C to allow for mycelial growth. Once the mycelium had completely covered the surface of the medium, the cultures were used as inoculum for seedling inoculation.

### 2.2 Experimental design

The controlled-environment experiment was conducted out during the growing season (April to November) of 2023 at the experimental nursery (Sanqingyuan Station) of Beijing Forestry University. Each plastic pot (24.5 cm top diameter × 25 cm height × 18.5 cm base diameter) was filled with 6 kg sterilized experimental soil. Three morphologically similar two-year-old Mongolian pine seedlings (height: 30.2 ± 1.5 cm; basal diameter: 4.3 ± 0.3 mm) were transplanted into each pot. After a one-month acclimatization period, the prepared fungal inoculum was applied. The inoculum was prepared by homogenizing the fully colonized PD agar medium (25 °C, dark, 4 weeks) with sterile distilled water in a blender. The resulting suspension was adjusted to a standard concentration of approximately 1.0 g fresh mycelial mass per 10 mL of sterile water. For each pot, a consistent volume of 50 mL of this inoculum suspension was evenly sprayed onto the soil surface around the seedling base. Control (CK) pots received the same volume of a sterile PD medium suspension prepared identically but without fungal biomass. To secure the inoculum and minimize desiccation, the treated soil surface was immediately covered with 600 g of fresh, sterilized substrate. The inoculation treatments included three experimental groups: (1) single inoculation with *Rhizopogon* sp. (Rh), (2) single inoculation with *Tomentella* sp. (To), and (3) dual inoculation with both fungal species (Rh+To). The control group (CK) received the same volume of sterile culture solution instead of ectomycorrhizal inoculum. Stringent spatial isolation (≥100 cm between treatment groups) was implemented to prevent airborne cross-contamination, seedlings exhibiting >80% ectomycorrhizal colonization were selected for the drought stress and re-watering experiments, with 20 biological replicates established for each treatment group.

The experiment established five progressive drought stress durations: starting with a control group (0 d) and continuous drought for 7 d, 14 d, 21 d, and 35 d of continuous drought. These durations corresponded to soil water contents of 22.16% (CK0, well-watered control), 11.50% (D7, mild drought), 4.85% (D14, moderate drought), 2.67% (D21, severe drought), and 1.42% (D35, extreme drought), which represent 80%, 46%, 19%, 11%, and 6% of the soil’s saturated water holding capacity (25%), respectively. Concurrently, a re-watering treatment group (R0-R35) was created, where the stressed seedlings were rewatered to initial well-watered conditions (80% of saturated water content). Physiological measurements were taken from five randomly chosen Mongolian pine seedlings for each treatment at two different times: right after the drought ended and 24 hours after re-watering.

### 2.3 Mycorrhizal colonization assessment

For the analysis of mycorrhizal colonization, five acclimatized Mongolian pine seedlings were randomly chosen for each treatment. The root systems were thoroughly washed, and ten 1-cm segments of roots were randomly selected from each seedling. The rates of colonization were measured using trypan blue staining (Giovannetti and Mosse, 1980). The specific calculation formula is given in Equation (1). The results showed that the Mongolian pine seedlings were effectively colonized by two species of EMF (see **Table 1**).

**Table 1:** Colonization rates of Mongolian pine ectomycorrhizal seedlings.

Treatment	CK	Rh	To	Rh+To
Colonization rates (%)	0	85.19 ± 5.34	89.48 ± 6.48	91.53 ± 6.14

CK, Uninoculated; Rh, *Rhizopogon* sp.; To, *Tomentella* sp.; Rh+To, Co-inoculation with *Rhizopogon* sp. and *Tomentella* sp.

(1) $$\text{Colonization rates}=\frac{\text{Number of mycorrhizal root segments}}{\text{Total number of root segments}}\times 100\text{\% }$$

### 2.4 Physiological measurements of seedlings

#### 2.4.1 Photosynthetic fluorescence measurements

Leaf photosynthetic parameters were assessed using a Li-6800 portable photosynthesis system (Li-COR, Lincoln, USA) between 09:00 and 11:00 on sunny days. The parameters measured included net photosynthetic rate (Pn), stomatal conductance (Gs), intercellular CO_2_ concentration (Ci), and transpiration rate (Tr), with data gathered from three different leaf positions (upper, middle, and lower canopy). After the measurements, the leaves were cut to determine leaf area for normalizing the photosynthetic parameters. Chlorophyll fluorescence parameters were measured using a Fluor Cam open fluorescence imaging system (Photon Systems Instruments, Drasov, Czech Republic) between 18:00 and 22:00 h. Prior to measurements, the plants were kept in the dark for 20 min. The parameters recorded included minimal fluorescence (F_0_), maximum photochemical efficiency of PSII (F_v_/F_m_), non-photochemical quenching (NPQ), and photochemical quenching coefficient (qP). For each treatment, five randomly chosen plants were analyzed, with three replicate measurements taken for each leaf.

#### 2.4.2 Water status parameters measurements

Leaf relative water content (RWC) and tissue density (TD) were assessed according to the methods by Barrs and Weatherley (1962). Leaf water use efficiency (WUE) was determined by calculating the ratio of the net photosynthetic rate (Pn) to the transpiration rate (Tr). Leaf (ψ_l_) and root (ψ_r_) water potentials were measured using a PSYPRO dew point potentiometer (Wescor, Logan, UT, USA). For each treatment, five randomly chosen seedlings were analyzed, with three replicate measurements taken for each leaf. The specific calculation formula is given in Equations (2)–(4).

(2) $$\text{Leaf relative water content }\left(\text{RWC}\right)=\frac{\text{Fresh weight }-\text{ Dry weight }}{\text{Dry weight }}\times 100\text{\% }$$

(3) $$\text{Leaf tissue Density }\left(\text{TD}\right)=\frac{\text{Dry weight}}{\text{Full turgidity weight}}\times 100\text{\% }$$

(4) $$\text{Leaf water use efficiency }\left(\text{WUE}\right)=\frac{\text{Pn}}{\text{Tr}}\times 100\text{\% }$$

#### 2.4.3 Leaf antioxidant capacity and osmolyte accumulation measurements

Leaf relative electrical conductivity (REC) was assessed using a pH meter (PHS-3E, LEICI, China) via the immersion technique (Li, 2002). The activities of leaf superoxide dismutase (SOD), catalase (CAT), and antioxidant enzyme (Peroxidase, POD), along with the levels of malondialdehyde (MDA) and proline (PRO) were measured using a spectrophotometer, Soluble sugar (SS) content was determined through aziridinium blue tetrazolium photoreduction, UV spectrophotometry, guaiacol colorimetry, thiobarbituric acid, ninhydrin colorimetry and anthrone colorimetry, respectively (Gao, 2006).

#### 2.4.4 Drought resistance, drought recovery and drought adaptability analysis

According to Chen et al. (2016), the evaluation of plant drought resistance involved measuring relative growth during periods of drought stress, while drought recovery capacity was assessed by observing regrowth after re-watering. Drought adaptability was determined by the overall growth performance during both stress and recovery stages.

### 2.5 Statistical analysis

All data presented as means ± standard error (SE). Statistical analyses were performed using SPSS 22.0 (IBM Corp, Armonk, NY, USA). A one-way ANOVA followed by Duncan’s multiple range test (*p* < 0.05) was used to evaluate the effects of treatments on physiological parameters. Two-way analysis of variance was employed to examine the effects of drought-rewatering, EMF inoculation, and their interaction on physiological indicators of seedlings at 0.05 significance level. Linear regression analyses of drought resistance, drought recovery, and drought adaptability indices were performed using OriginPro 8.0 (OriginLab, Northampton, MA, USA). Multivariate analyses in R 4.3.0 (R Foundation, Vienna, Austria) included principal component analysis (PCA) of stress and re-watering responses, correlation-based cluster analysis, and Pearson correlation matrices linking physiological traits with drought performance indices, with results visualized as PCA biplots and clustered heatmaps.

## 3 Results

### 3.1 Drought and re-watering alterations in leaf gas exchange and fluorescence dynamics in ectomycorrhizal seedlings of Mongolian pine

**Figure 1:**
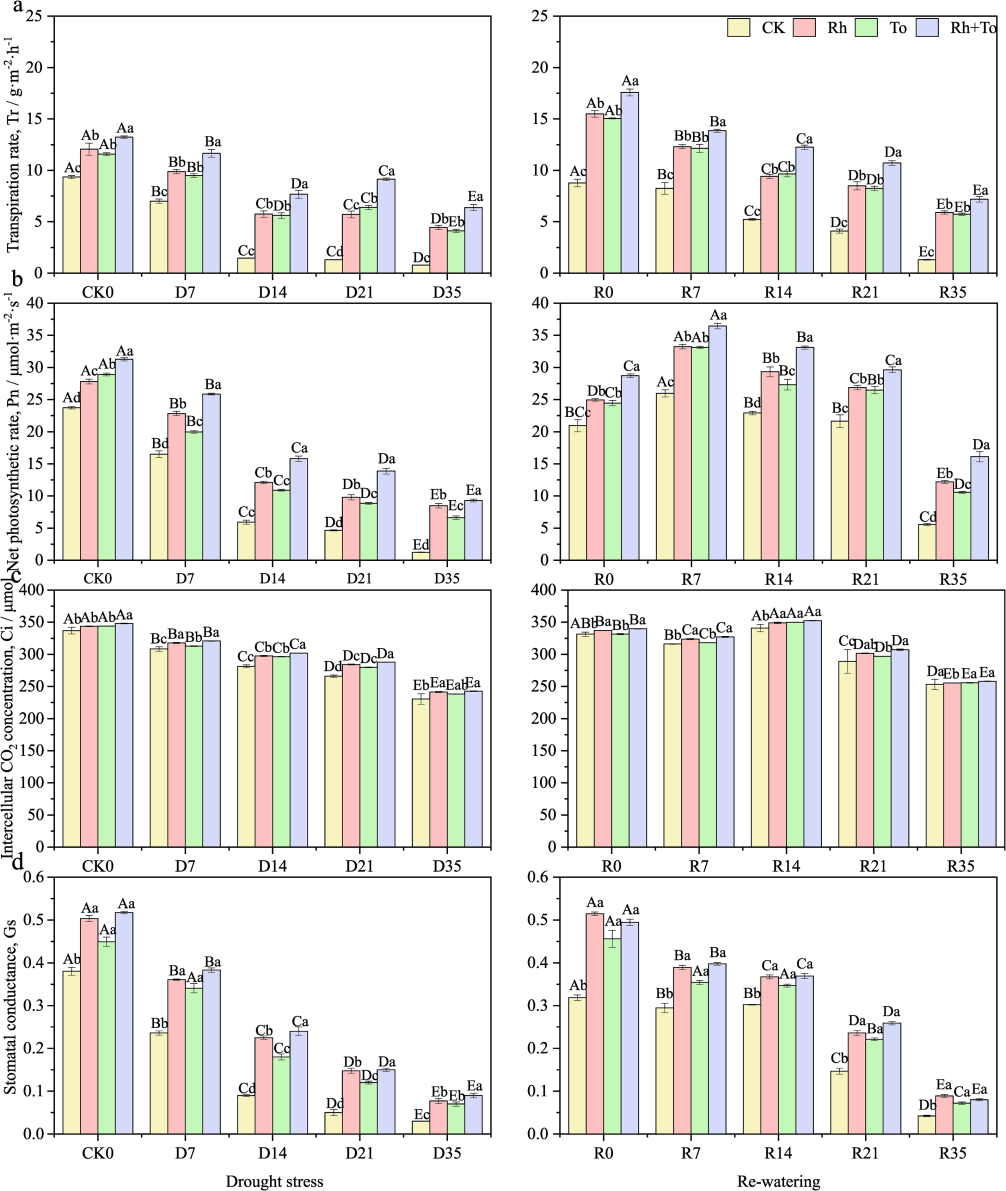
Effect of drought and re-watering on leaf photosynthetic parameters of Mongolian pine seedlings. [**(A)** transpiration rate, **(B)** net photosynthetic rate, **(C)** intercellular CO_2_ concentration, **(D)** stomatal conductance]. Different capital letters indicate significant difference among the different moisture treatments in the same inoculation treatment; the different small letters indicate significant difference among the different inoculation treatments in the moisture treatment, the same below.

**Figure 2:**
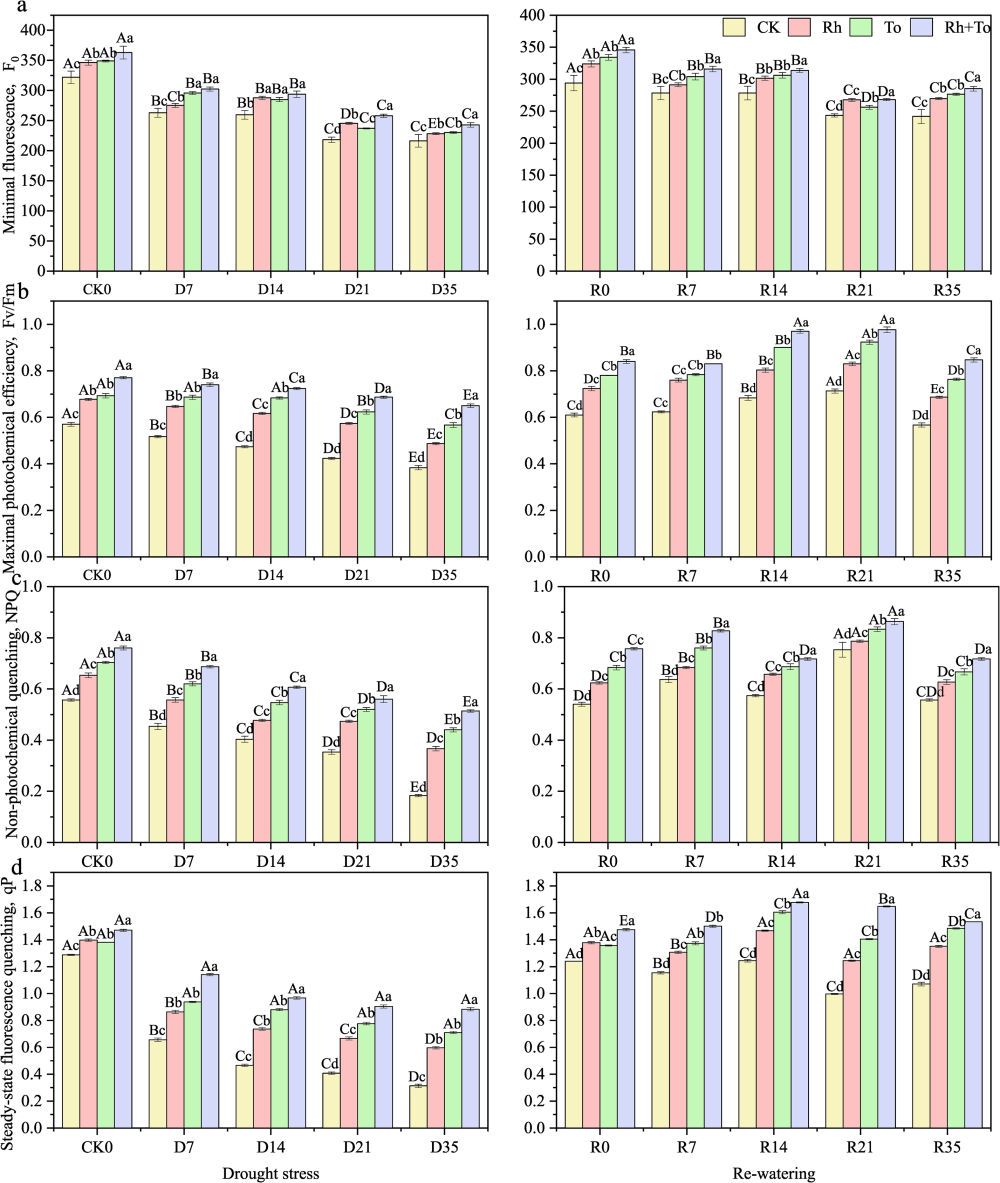
Effect of drought and re-watering on leaf fluorescence parameters of Mongolian pine seedlings. [**(A)** minimal fluorescence, **(B)** maximal photochemical efficiency, **(C)** non-photochemical quenching, **(D)** steady-state fluorescence quenching].

Drought stress, re-watering, and ECM inoculation had exerted highly significant impact on the photosynthetic and chlorophyll fluorescence characteristics of seedlings (*P* < 0.01). The contribution of drought-rewatering cycles to parameter variation exceeded that of ECM inoculation. A significant interaction between drought-rewatering and ECM inoculation was observed only for Ci and qP (*P* < 0.01, **Supplementary Table 1**). Drought stress significantly impaired both photosynthetic and photochemical efficiency in Mongolian seedlings (*P* < 0.05). Tr, Pn, Ci, Gs, F_o_, Fv/Fm, NPQ, and qP all progressively declined with increasing stress intensity (**Figures 1**, **2**), reaching the lowest levels under D35 (*P* < 0.01, vs. CK). Following re-watering, all parameters recovered to varying, stress−dependent degrees, with rewatered seedlings consistently outperforming those kept under continuous drought. ECM inoculation significantly improved photosynthetic performance and photochemical efficiency during both drought and the following re-watering (*P* < 0.05; **Figures 1**, **2**). Co−inoculation (Rh+To) yielded better photosynthetic results than singly inoculations, with Rh>To for photosynthetic parameters and To>Rh for fluorescence traits.

### 3.2 Drought and re-watering alterations in water physiological dynamics in ectomycorrhizal seedlings of Mongolian pine

**Figure 3:**
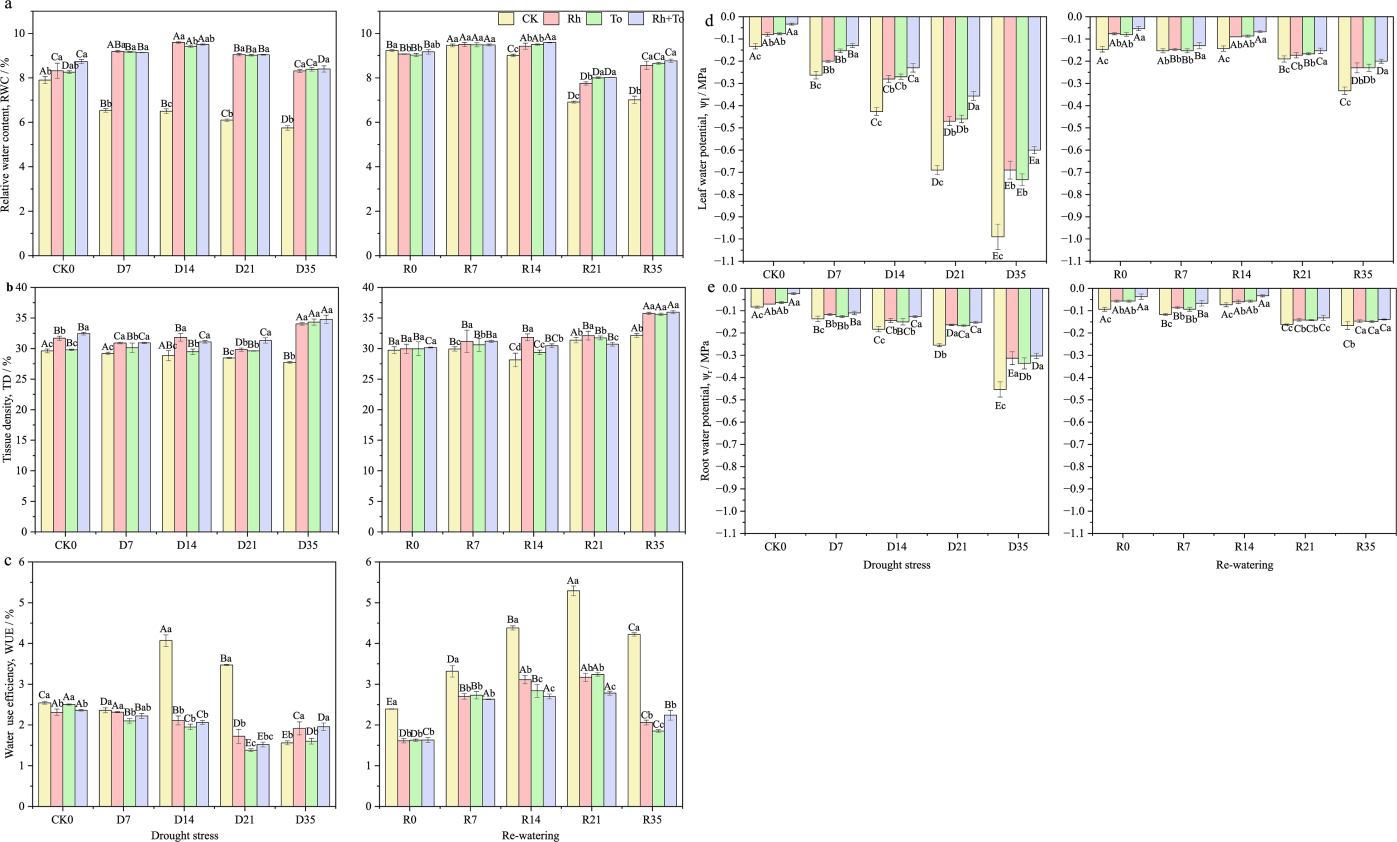
Effect of drought and re-watering on water physiology parameters of Mongolian pine seedlings. [**(A)** relative water content, **(B)** tissue density, **(C)** water use efficiency, **(D)** leaf water potential, **(E)** root water potential].

Drought, re-watering, and ECM inoculation each exerted highly significant effects on water−relation traits of seedlings (*P* < 0.01). Changes in RWC, TD and WUE were influenced more strongly by fungal inoculation, whereas variations in ψ_l_ and ψ_r_ were driven mainly by soil−moisture cycles (*P*>0.05, **Supplementary Table 2**). Under drought stress, ψ_l_ and ψ_r_ decreased significantly (*P* < 0.05, **Figures 3D, E**), in RWC and TD also declined in uninoculated seedlings as stress intensified (*P* < 0.05, **Figures 3A, B**). After re-watering, seedling water relations were restored differently depending on the intensity of drought stress. Both WUE and ψ_r_ were significantly higher in rewatered seedlings compared to well-watered controls (*P* < 0.05) (**Figures 3C, E**). ECM inoculation significantly improved most water-relation parameters after re−watering under severe drought (*P* < 0.05), expect for WUE and TD. Co-inoculated seedlings consistently exhibited higher ψ_l_ and ψ_r_ than singly inoculated group.

### 3.3 Drought and re-watering alterations in osmotic adjustment and antioxidant ability dynamics in ectomycorrhizal seedlings of Mongolian pine

**Figure 4:**
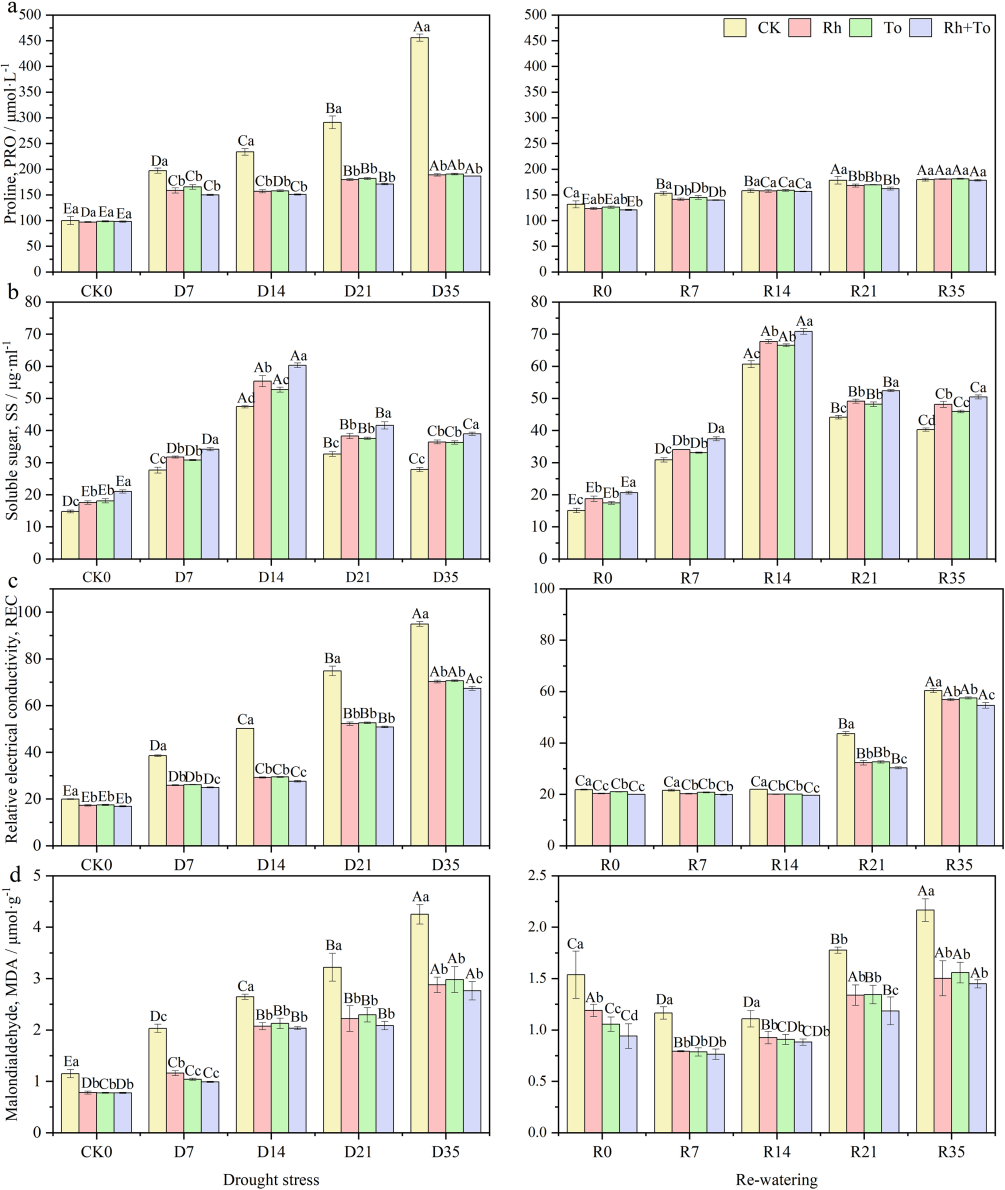
Effect of drought and re-watering on osmoregulation capacity of Mongolian pine seedlings. [**(A)** proline, **(B)** soluble sugar, **(C)** relative electrical conductivity, **(D)** malondialdehyde].

**Figure 5:**
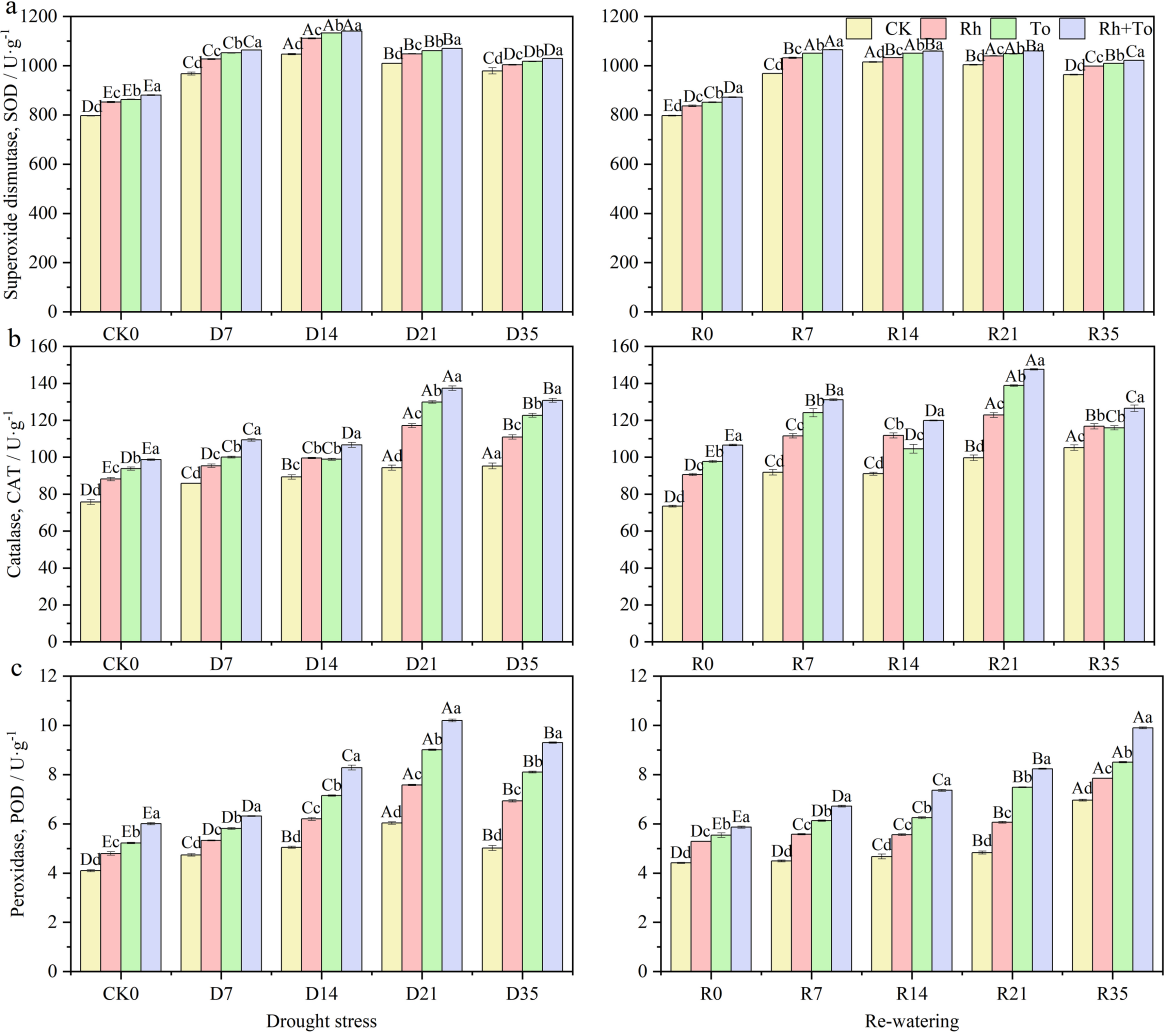
Effect of drought and re-watering on antioxidant enzyme activities of Mongolian pine seedlings. [**(A)** superoxide dismutase, **(B)** catalase, **(C)** peroxidase].

Drought, re-watering, and ECM inoculation each significantly influenced osmoregulatory substances, cell membrane stability, and antioxidant enzyme activities (*P* < 0.01), with drought-rewatering cycles accounting for a greater share of the variation than ECM inoculation. Their interaction was significant only for Pro, MAD, and CAT (*P* < 0.01, **Supplementary Table 2**). Drought stress markedly elevated REC, Pro, SS, and MDA content, as well as the activities of SOD, CAT, and POD (*P* < 0.05, **Figure 4**). Pro and MDA content, as well as REC, rose progressively with increasing drought intensity (*P* < 0.05). After re-watering, Pro and REC declined relative to drought−stressed seedlings but remained above well-watered control levels. By contrast, SS content and antioxidant enzyme activities after re−watering were significantly higher compared to the well-watered treatment (*P* < 0.05). ECM inoculation reduced Pro and MDA contents and REC, while increasing SS accumulation. All three antioxidant enzymes activities were significantly higher in ectomycorrhizal seedlings than in non-inoculated controls (*P* < 0.05), and co-inoculation further enhanced these activities compared with single inoculation(*P* < 0.05) (**Figure 5**).

### 3.4 Drought resistance, drought recovery, and drought adaptation of Mongolian pine ectomycorrhizal seedlings

**Figure 6:**
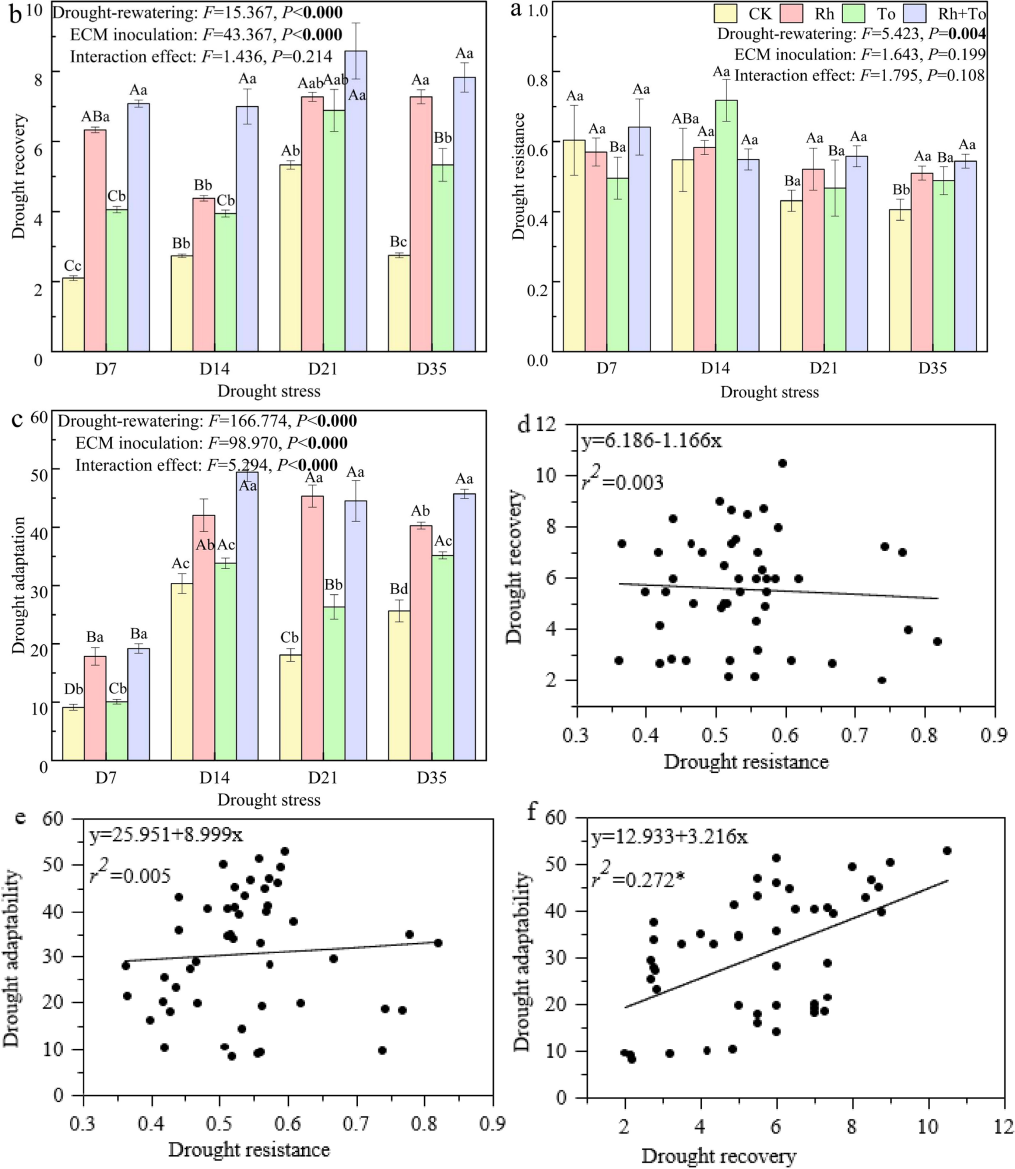
Drought resistance, drought recovery and drought adaptability and their correlations of Mongolian pine seedlings under drought and re-watering conditions.

**Figure 7:**
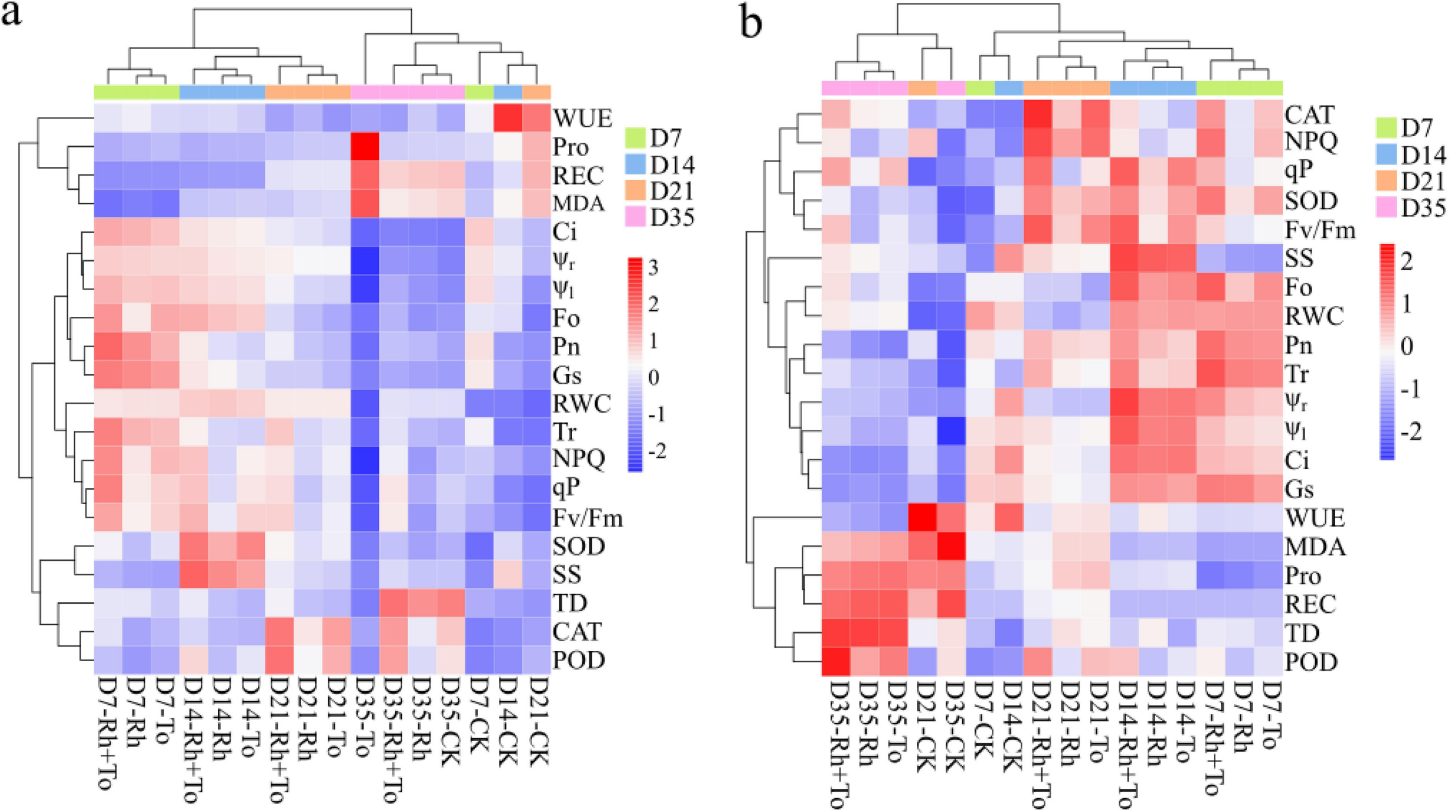
Corheatmap analysis of biochemical indicators of Mongolian pine seedlings under drought **(A)** and re-watering **(B)** Note: different colors in the legend represent different drought stress and re-watering treatments; darker red represents stronger positive correlation and darker blue represents stronger negative correlation.

Drought and re-watering significantly influenced the drought resistance, drought recovery, and drought adaptation of seedlings (*P* < 0.01) (**Figure 6**). ECM inoculation notably enhanced drought recovery and adaptation capabilities (*P* < 0.01). Although drought stress decreased seedling drought resistance, it improved their drought recovery and drought adaptability (*P* < 0.05), with resistance declining as stress intensity increased. ECM inoculation generally increased all three performance indices (except under D7), with efficacy typically followed: dual inoculation > single Rh inoculation > single To inoculation, although drought resistance under D14 varied from this order. Linear regression revealed weak correlations between drought resistance and recovery capacity (*r*²=0.003) or drought adaptability (*r*²=0.005, **Figure 7**), whereas drought recovery capacity and drought adaptability were significantly correlated (*r*² = 0.272^*^).

### 3.5 Response of Mongolian pine ectomycorrhizal seedlings to drought-rewatering

**Figure 8:**
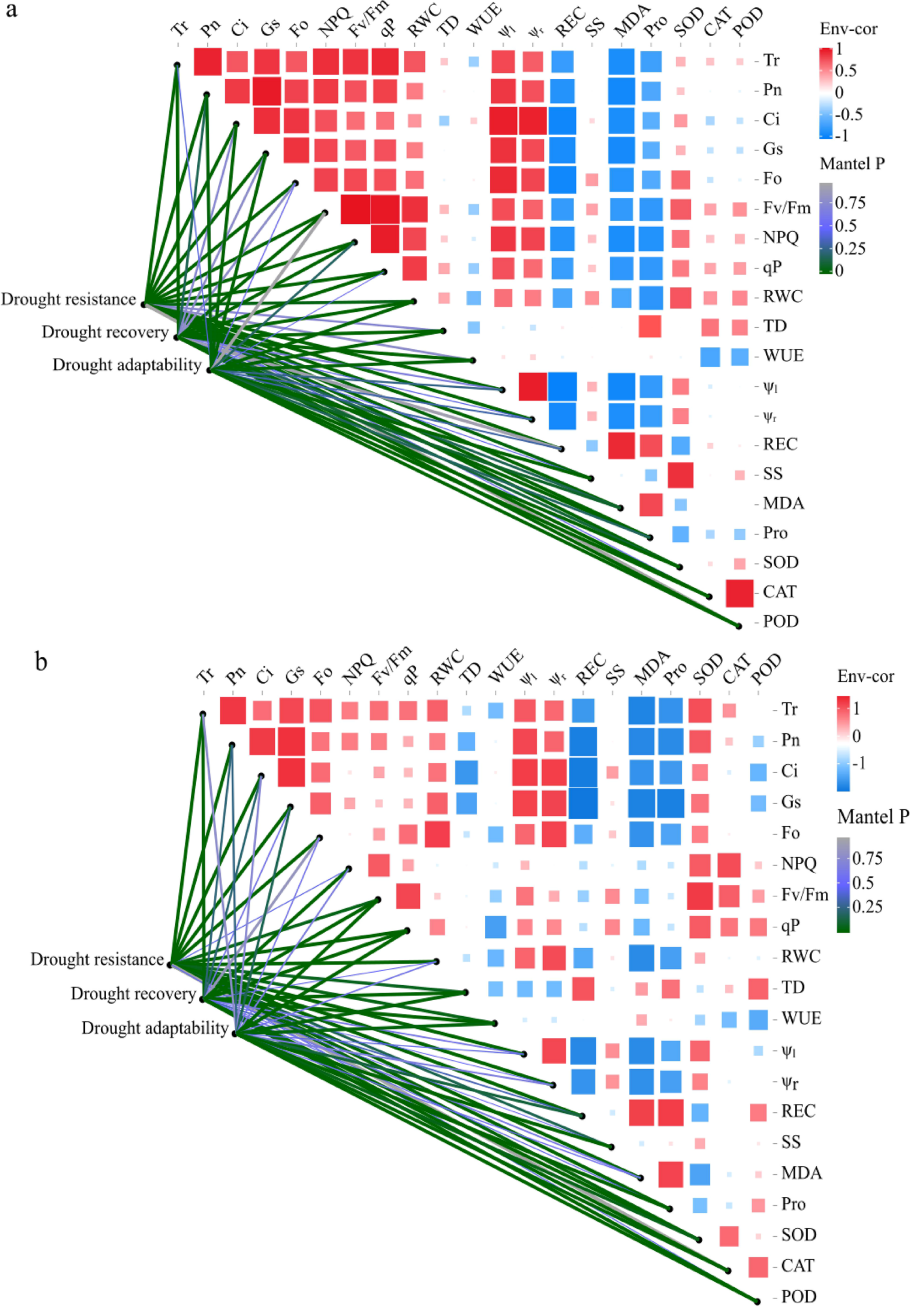
Correlations between the biochemical indicators and drought resistance, drought recovery, drought adaptability of Mongolian Pine seedlings under drought **(A)** and re-watering **(B)** conditions.

PCA revealed that under drought conditions, PC1 and PC2 accounted for 59.04% and 20.3% of the total variance, respectively (cumulative 79.34%). After re-watering, the explained variances were 53.09% (PC1) and 22.18% (PC2), respectively, totaling 75.27%. During drought, samples from various stress-intensity showed scattered distribution, whereas after re-watering, samples from D7 and D14 clustered more closely, while D21 and D35 treatments remained separated (**Supplementary Figure S1**; **Figure 7**). Notably, seedlings receiving the same EMF inoculation consistently grouped together. Cluster analysis further revealed that seedling water potential, WUE, osmotic regulating substances and cell membrane stability exhibited similar trends, while photosynthetic characteristics, fluorescence parameters and RWC showed comparable patterns (**Figure 8**). Correlations among physiological characteristics were generally weaker under drought than after re-watering.

Overall, physiological characteristics showed stronger correlations with drought resistance than with drought recovery or drought adaptability (**Figure 8**). During drought, drought resistance had significant (*P* < 0.05) or highly significant (*P* < 0.01) correlations with most parameters, except TD, WUE, CAT, and POD. Recover capacity correlated positively with Tr, RWC, TD, CAT, and POD (*P* < 0.01), but negative with WUE and Pro (*P* < 0.01). Drought adaptability was positively correlated with antioxidant enzyme activities (*P* < 0.01), and negatively to Pn, Ci, Gs, and WUE (*P* < 0.05). After re-watering, drought tolerance showed no significant correlation with NPQ, TD, SS, CAT or POD (*P*>0.05), whereas drought resilience was highly positively with Fv/Fm, NPQ, qP, and antioxidant enzyme activities (*P* < 0.01). Additionally, drought adaptation was positively associated with SS and REC (*P* < 0.01).

## 4 Discussion

### 4.1 Drought-rewatering effects on physiological responses in ectomycorrhizal seedlings of Mongolian pine

Under drought stress, plant growth is regulated by a sophisticated network of physiological, biochemical, and molecular processes, often initiated by morphological adjustments that trigger subsequent metabolic responses (Martínez-Vilalta & Garcia-Forner, 2016; Zhang et al., 2022). Consistent with our first hypothesis, the present study demonstrates that drought stress led to a progressive decline in photosynthetic performance, chlorophyll fluorescence, and water status of Mongolian pine seedlings. We observed pronounced, stress-intensity-dependent reductions in key parameters, including Pn, Gs, F_0_, Fv/Fm, NPQ, qP, ψ_l_, ψ_r_, and RWC, consistent with known drought response patterns in conifers, wherein photosynthesis is particularly vulnerable to water deficit (Jia et al., 2021). As the principal route for carbon assimilation and a central regulator of drought responses, photosynthesis is highly sensitive to limited water availability (Li et al., 2018). The reduction in Gs and Tr represents a water-conserving strategy that lowers tissue water potential, albeit at the cost of restricted CO_2_ uptake and consequent depression of photosynthetic efficiency (Bellasio, 2023). Chlorophyll fluorescence provides critical insight into the photophysical processes occurring within chloroplasts. Concurrently, the decline in NPQ and qP under intensified drought indicates a substantial light-dependent suppression of photosynthesis and a diminished capacity for photoprotection and regulation of photosynthetic electron transport (Brestic and Zivcak, 2013). Alongside these photophysical changes, seedlings activated several acclimation mechanisms, including the accumulation of osmolytes (such as Pro and SS) for osmotic adjustment, reduction in RWC and TD to enhance water conservation, and elevation of antioxidant enzyme activities to scavenge reactive oxygen species and preserve membrane integrity (Gupta et al., 2020). Collectively, these responses from a multi-layered defense strategy that enhances seedling resilience under drought, yet they were insufficient to prevent a progressive physiological deterioration as stress intensity increased.

The second hypothesis, that EMF inoculation would significantly mitigate the drought-induced physiological impairments, was strongly supported by our data. Inoculated seedlings, particularly those with dual inoculation (Rh+To), consistently maintained higher photosynthetic parameters, chlorophyll fluorescence indices, and water-relation metrics under drought stress compared to non-inoculated controls. This protective effect can be attributed to several mechanisms mediated by the fungal symbionts, which enhance drought tolerance through improved photosynthesis, osmoregulation, and antioxidant capacity (Ren et al., 2023; Liu et al., 2021). Specifically, EMF symbionts helped sustain photosynthetic efficiency under drought by alleviating the decline in gas exchange and chlorophyll fluorescence, thereby supporting stable photosynthetic performance. This ensures that, under drought conditions, ECM are critical for Mongolian pine seedlings to sustain stable and efficient photosynthetic performance. In terms of water regulation, EMF enhanced water uptake efficiency through modifications in root system architecture and improved osmoregulatory function in root cells, which collectively contributed to maintaining higher leaf relative water content (Lehto and Zwiazek, 2011; Castaño et al., 2023). Furthermore, in antioxidant defense, ECM effectively mitigate oxidative damage and protect cellular membrane systems by increasing the MDA, POD, CAT and SOD activities in seedlings (Madouh and Quoreshi, 2023). The observed resilience underscores the multifaceted nature of the EMF–plant symbiosis in stress adaptation. Beyond improving nutrient access, EMF enhance drought resilience through integrated mechanisms that include: (i) extending the functional root volume via extraradical mycelium, thereby improving water foraging in drying soil; (ii) potentially facilitating hydraulic redistribution or moderating water transport resistance; (iii) priming the host’s antioxidant and osmotic adjustment systems; and (iv) modulating stress-related phytohormone signaling. The superior performance of dual inoculation suggests potential synergistic or complementary effects between *Rhizopogon* sp. and *Tomentella* sp. in supporting the host plant under drought stress.

Following post-drought rewatering, plants activate intrinsic self-regulatory and compensatory mechanisms that drive physiological recovery, the extent of which is largely governed by the severity of the preceding drought (Khoyerdi et al., 2016; Liao et al., 2018). Consistent with our third hypothesis, EMF-inoculated seedlings displayed accelerated and more complete physiological recovery compared to non-inoculated controls, with the extent of recuperation closely tied to prior stress intensity. Under mild drought (D7), chlorophyll fluorescence parameters (F_0_, Fv/Fm, NPQ, qP) and photosynthetic traits (Pn, Tr, Ci, Gs) in EMF-inoculated seedlings rebounded to levels matching or even exceeding those of well-watered controls, indicating reversible PSII impairment and active compensatory responses. In contrast, seedlings subjected to extreme drought (D35) exhibited incomplete recovery, reflecting sustained photochemical and physiological dysfunction induced by intense water deficit (Qi et al., 2021). These results highlight that EMF enhance not only drought tolerance but also the regenerative capacity of Mongolian pine seedlings during rewatering, especially after moderate stress.

Optimal plant water status, reflected by RWC, plays a critical role in maintaining physiological function and enhancing drought resilience (Cai et al., 2020). Leaf dehydration is usually a reversible process, and both RWC and TD of Mongolian pine seedlings recovered significantly after rewatering. Plants restore growth and photosynthetic functionality following re-watering via mechanisms such as regenerative tissue proliferation, stomatal reopening, and repair of oxidative damage. However, recovery efficiency depends on stress intensity, duration, and species-specific drought tolerance thresholds (Li et al., 2020). Furthermore, rewatering significantly reduces of osmoregulatory substances (Pro, SS and MDA) and the activities of oxidative enzymes (CAT, SOD and POD), thereby rapidly restoring cell membrane permeability and improving the capacity of the antioxidant system. These findings align with our study’s results, demonstrating that rewatering markedly improves osmoregulatory processes and cell membrane stability in Mongolian pine seedlings.

### 4.2 Response of Mongolian pine ectomycorrhizal seedlings to drought-rewatering stress

In ecologically fragile regions prone to frequent droughts and sandstorms, Mongolian pine plantations are routinely exposed to dynamic cycles of water stress and re-watering. These fluctuating moisture conditions significantly impair plant growth, photosynthetic function, and essential metabolic processes, ultimately threatening forest health and productivity. As a crucial dune-fixing afforestation species in northern China’s sandstorm-prone areas, the drought resistance, stress tolerance, and post-rewatering recovery capacity of Mongolian pine directly determine its ecological benefits. Conventional wisdom holds that plants can rapidly and fully recover to a healthy state after the alleviation of drought stress (Anderegg et al., 2015). However, recovery of plant physiological functions exhibits a gradual progression, and this process is critical for maintaining ecosystem stability. If recurrent drought stress occurs before full recovery is achieved, it may trigger irreversible regime shifts in ecosystems (Luo et al., 2015). Over the past decade, research on the drought recovery capacity of plants has received increasing scientific attention (Chen et al., 2016). Consequently, understanding drought impacts on plants requires not only examining physiological responses during stress, but also the systematic characterizing recovery dynamics after re-watering.

Plant responses to drought and rewatering involve coordinated physiological and biochemical mechanisms (Xu et al., 2010), which collectively shape plant drought tolerance, drought resilience, and drought adaptation. Our study revealed distinct physiological response patterns between drought resistance and drought adaptation capacities in Mongolian pine seedlings during drought-rewatering cycles. Critical physiological parameters showed contrasting correlations between the drought stress and the post-rewatering recovery. This finding confirms that the drought resistance and recovery capacity of plants are governed by distinct physiological mechanisms, with the stability of the photosynthetic fluorescence system playing a crucial role in facilitating rapid recovery after rewatering. PCA revealed that drought adaptation strategies varied with stress intensity. Under mild to moderate drought and during rehydration, photosynthetic parameters emerged as the primary determinants of drought tolerance. Under severe to extreme drought, however, osmolyte accumulation and antioxidant enzyme activities became critical. This gradient response profile illustrates that plants have constructed a multi-level regulatory mechanism to cope with cyclic drought stress through the integrated coordination of drought resistance, post-stress recovery capacity, and adaptive plasticity. Under drought-induced stress conditions, drought resistance, drought recovery, and drought adaptability serve as vital self-regulatory mechanisms for plants to cope with drought stress (Fang and Xiong, 2015). Linear regression analysis revealed that drought recovery capacity played a more dominant role than resistance in shaping overall drought adaptation in Mongolian pine. This suggests that under prolonged drought conditions, post-rewatering recovery capacity potentially holds greater ecological importance than mere drought resistance, providing new theoretical insights for understanding plant adaptation strategies to periodic drought stress.

Plant water status constitutes a critical determinant of growth and physiological performance, and maintaining a high RWC is strongly correlation with plant drought resistance (Chen et al., 2016; Bartoli et al., 1999). In this study, ψ_l_, ψ_r_, and RWC all exhibited significant to highly significant positive correlations with plant drought resistance. This confirms that water potential parameters and RWC serve as effective indicators for assessing plant water status. It also confirms that maintaining optimal hydration primarily improves drought resistance capacity rather than post-drought recovery. In response to drought stress, Mongolian pine seedlings employ a multifaceted strategy to enhance their drought resilience: (1) morphological adaptation, which involves root system proliferation, leaf area reduction, and modulation of stomatal aperture to optimize water uptake efficiency; (2) physiological regulation, which maintains cell turgor pressure through the accumulation of osmoregulatory substances such as Pro and SS; (3) cellular membrane system protection, which strengthens the stability of cellular membranes under drought stress; (4) antioxidant defense system, which enables efficient ROS scavenging through the dynamic modulation of enzymatic activities, including SOD, POD, and CAT. Consistent with this framework, drought stress significantly increased MDA content and antioxidant enzyme activities, whereas rewatering markedly decreased them. Correlation analysis further revealed that antioxidant enzyme activities were significantly positively correlated with drought adaptation capacity during drought stress (*P* < 0.05). In contrast, these activities were more strongly associated with drought recovery capability during the rewatering phase (*P* < 0.05). Based on these findings, we propose that MDA content and antioxidant enzyme activities serve as reliable physiological indicators for assessing drought resistance in Mongolian pine seedlings. This approach provides a crucial theoretical foundation for developing a species-specific drought resistance evaluation system.

This study establishes a physiological foundation for improving drought tolerance in Mongolian pine, a keystone species for ecological restoration in arid and semi-arid regions. Our findings point to two main directions for future research. First, the marked resilience conferred by dual-fungal inoculation (Rh+To) underscores the potential of optimized ectomycorrhizal partnerships; subsequent work should employ transcriptomic and metabolomic approaches to unravel the molecular dialogue and functional complementarity underlying this synergistic effect. Second, to translate these controlled-environment results into practical restoration strategies, field trials are needed to evaluate the long-term survival and growth of inoculated seedlings under natural, fluctuating drought regimes.

## 5 Conclusion

Drought stress has detrimental impacts on the physiological performance of Mongolian pine seedlings. This study demonstrated that EMF inoculation and rewatering treatments effectively alleviated drought-induced damage through several synergistic mechanisms. Firstly, by maintaining higher respiration rates and enhancing photosynthetic efficiency (Tr increased by 40.72% to 298.02%), this approach mitigated the drought-induced inhibition of photosystem II functionality (NPQ elevated by 18.03% to 211.11%). Secondly, it significantly improved leaf water status, as evidenced by an increase in relative water content (RWC upregulation of 2.05% to 45.57%) and enhanced water retention capacity (TD improvement from 1.12% to 16.63%), thereby promoting hydraulic transport efficiency (ψr enhancement of 14.29% to 76.52%). Furthermore, through the coordinated regulation of osmotic adjustment substances (soluble sugar accumulation ranging from 5.00% to 47.22%) and modulation of antioxidant enzyme activities (SOD and POD activities reduced by 0.31% to 7.21% and 9.07% to 20.76%, respectively), cellular membrane stability was preserved (MDA reduction ranging from 16.75% to 62.73%), culminating in significantly enhanced physiological adaptability to drought stress.

Following re-watering, Mongolian pine seedlings exposed to varying intensities of drought exhibited distinct physiological compensatory responses. Overall, seedlings under mild drought stress demonstrated enhanced compensatory effects, while those subjected to extremely severe drought stress showed a significantly reduced capacity for recovery. Under drought-rewatering conditions, Mongolian pine ectomycorrhizal seedlings exhibited improved drought resistance, superior post-stress recovery capacity, and increased adaptive resilience. The mechanisms underlying their responses to drought-rewatering can be summarized as follows: EMF synergistically enhanced seedling drought tolerance through coordinated physiological improvements, including (1) promoting photosynthetic performance, (2) optimizing foliar water status, (3) regulating osmotic homeostasis in mesophyll cells, and (4) scavenging ROS damage, thereby establishing a multi-layered drought defense system.

## Data Availability

The original contributions presented in the study are included in the article/**Supplementary Material**. Further inquiries can be directed to the corresponding authors.

## References

[B1] Abid M. Ali S. Qi L. K. Zahoor R. Dai T. (2018). Physiological and biochemical changes during drought and recovery periods at tillering and jointing stages in wheat (*Triticum aestivum* L.). Sci. Rep. 8, 4615. doi: 10.1038/s41598-018-21441-7, PMID: 29545536 PMC5854670

[B2] Agurla S. Gahir S. Munemasa S. Murata Y. Raghavendra A. S. (2018). Mechanism of stomatal closure in plants exposed to drought and cold stress. Adv. Exp. Med. Biol. 1081, 215–232. doi: 10.1007/978-981-13-1244-1_12, PMID: 30288712

[B3] Anderegg W. R. Schwalm C. Biondi F. Camarero J. J. Koch G. Litvak M. . (2015). Pervasive drought legacies in forest ecosystems and their implications for carbon cycle models. Science 349, 528–532. doi: 10.1126/science.aab1833, PMID: 26228147

[B4] Anjum S. A. Ashraf U. Zohaib A. Tanveer M. (2017). Growth and development responses of crop plants under drought stress: A review. Zemdirbyste 104, 267–276. doi: 10.13080/z-a.2017.104.034

[B5] Barrs H. D. Weatherley P. E. (1962). A re-examination of the relative turgidity technique for estimating water deficits in leaves. Aus. J. Biol. Sci. 15, 413–428. doi: 10.1071/bi9620413

[B6] Bartoli C. G. Simontacchi M. Tambussi E. Beltrano J. Montaldi E. Puntarulo S. (1999). Drought and watering-dependent oxidative stress: effect on antioxidant content in *Triticum aestivum* L. leaves. J. Exp. Bot. 50, 375–385. doi: 10.1093/jxb/50.332.375

[B7] Bellasio C. (2023). The slope of assimilation rate against stomatal conductance should not be used as a measure of water use efficiency or stomatal control over assimilation. Photosynth. Res. 158, 195–199. doi: 10.1007/s11120-023-01054-6, PMID: 37902923 PMC10695868

[B8] Brestic M. Zivcak M. (2013). “ PSII fluorescence techniques for measurement of drought and high temperature stress signal in crop plants: protocols and applications,” in Molecular Stress Physiology of Plants. Eds. Rout G. Das A. ( Springer Press, Orissa, India). doi: 10.1007/978-81-322-0807-5_4

[B9] Bogati K. Walczak M. (2022). The impact of drought stress on soil microbial community, enzyme activities and plants. Agronomy. 12, 189–203. doi: 10.3390/agronomy12010189

[B10] Cai F. Zhang Y. S. Mi N. Ming H. Q. Zhang S. J. Zhang H. . (2020). Maize (*Zea mays* L.) physiological responses to drought and rewatering, and the associations with water stress degree. Agr. Water Manage. 241, 106379. doi: 10.1016/j.agwat.2020.106379

[B11] Castaño C. Suarez-Vidal E. Zas R. Bonet J. A. Oliva J. Sampedro L. (2023). Ectomycorrhizal fungi with hydrophobic mycelia and rhizomorphs dominate in young pine trees surviving experimental drought stress. Soil Biol. Biochem. 178, 108932. doi: 10.1016/j.soilbio.2022.108932

[B12] Chen D. Q. Wang S. W. Cao B. B. Cao D. Leng G. H. Li H. B. . (2016). Genotypic variation in growth and physiological response to drought stress and re-watering reveals the critical role of recovery in drought adaptation in Maize seedlings. Front. Plant Sci. 6. doi: 10.3389/fpls.2015.01241, PMID: 26793218 PMC4709455

[B13] Chen T. Zhu C. C. Li S. C. Xia Y. Huang J. Wang W. . (2025). Impact of ectomycorrhizal symbiosis on root system architecture and nutrient absorption in Chinese chestnut and pecan seedlings. Plant Soil 34, 1. doi: 10.1007/s11104-025-07332-7

[B14] de Mesquita C. P. B. Solon A. J. Barfield A. Mastrangelo C. F. Tubman A. J. Vincent K. . (2023). Adverse impacts of roundup on soil bacteria, soil chemistry and mycorrhizal fungi during restoration of a Colorado grassland. Appl. Soil Ecol. 185, 104778. doi: 10.1016/j.apsoil.2022.104778

[B15] Deng J. F. Yao J. Q. Zheng X. Gao G. L. (2021). Transpiration and canopy stomatal conductance dynamics of Mongolian pine plantations in semiarid deserts, Northern China. Agr. Water Manage. 249, 106806. doi: 10.1016/j.agwat.2021.106806

[B16] Fang Y. Xiong L. (2015). General mechanisms of drought response and their application in drought resistance improvement in plants. Cell. Mol. Life Sci. 72, 673–689. doi: 10.1007/s00018-014-1767-0, PMID: 25336153 PMC11113132

[B17] Gao J. F. (2006). Plant physiology experimental guidance (Beijing: Higher Education Press).

[B18] Giovannetti M. Mosse B. (1980). An evaluation of techniques for measuring vesicular arbuscular mycorrhizal infection in roots. New Phytol. 84, 489–500. doi: 10.1111/j.1469-8137.1980.tb04556.x

[B19] Gupta A. Rico-Medina A. Caño-Delgado A. I. (2020). The physiology of plant responses to drought. Science 368, 266–269. doi: 10.1126/science.aaz7614, PMID: 32299946

[B20] Jia Z. Lin T. Guo X. Zheng Y. Geng H. Zhang J. . (2024). Vegetation greening mitigates the positive impacts of climate change on water availability in Northwest China. J. Hydrol. 644, 132086. doi: 10.1016/j.jhydrol.2024.132086

[B21] Jia P. Melnyk A. Zhang Z. Li L. Kong X. Dai H. . (2021). Effects of drought and rehydration on the growth and physiological characteristics of mustard seedlings. J. Cent. Eur. Agric. 22, 836. doi: 10.5513/JCEA01/22.4.3246

[B22] Khoyerdi F. F. Shamshiri M. H. Estaji A. (2016). Changes in some physiological and osmotic parameters of several pistachio genotypes under drought stress. Sci. Hortic-Amsterdam 198, 44–51. doi: 10.1016/j.scienta.2015.11.028

[B23] Lehto T. Zwiazek J. J. (2011). Ectomycorrhizas and water relations of trees: A review. Mycorrhiza 21, 71–90. doi: 10.1007/s00572-010-0348-9, PMID: 21140277

[B24] Li H. S. (2002). Modern Plant Physiology (Beijing: Higher Education Press).

[B25] Li L. J. Gu W. R. Li J. Li C. F. Xie T. L. Qu D. Y. . (2018). Exogenously applied spermidine alleviates photosynthetic inhibition under drought stress in Maize (*Zea mays* L.) seedlings associated with changes in endogenous polyamines and phytohormones. Plant Physiol. Bioch. 129, 35–55. doi: 10.1016/j.plaphy.2018.05.017, PMID: 29793181

[B26] Li S. Wan L. Q. Nie Z. N. Li X. L. (2020). Fractal and topological analyses and antioxidant defense systems of Alfalfa (*Medicago sativa* L.) root system under drought and rehydration regimes. Agronomy. 10, 805. doi: 10.3390/agronomy10060805

[B27] Liao T. Wang Y. Xu C. P. Li Y. Kang X. Y. (2018). Adaptive photosynthetic and physiological responses to drought and rewatering in triploid *Populus* populations. Photosynthetica 56, 578–590. doi: 10.1007/s11099-017-0704-5

[B28] Liu S. X. Wang H. W. Qin F. (2023). Genetic dissection of drought resistance for trait improvement in crops. Crop J. 11, 975–985. doi: 10.1016/j.cj.2023.05.002

[B29] Liu N. Zhao Z. Y. Jiang X. L. Xing X. K. (2021). Review and prospect of researches on the mechanisms of mycorrhizal fungi in improving plant drought resistance. Mycosystema 40, 851–872. doi: 10.13346/j.mycosystema.200370

[B30] Lu Y. Duursma R. A. Farrior C. E. Medlyn B. E. Feng X. (2019). Optimal stomatal drought response shaped by competition for water and hydraulic risk can explain plant trait covariation. New Phytol. 225, 1206–1217. doi: 10.1111/nph.16207, PMID: 31538667

[B31] Luo Y. Keenan T. F. Smith M. (2015). Predictability of the terrestrial carbon cycle. Global Change Biol. 21, 1737–1751. doi: 10.1111/gcb.12766, PMID: 25327167

[B32] Madouh T. A. Quoreshi A. M. (2023). The function of arbuscular mycorrhizal fungi associated with drought stress resistance in native plants of arid desert ecosystems: A review. Diversity 15, 391. doi: 10.1111/nph.17692, PMID: 34449895

[B33] Martínez-Vilalta J. Garcia-Forner N. (2016). Water potential regulation, stomatal behaviour and hydraulic transport under drought: deconstructing the iso/anisohydric concept. Plant Cell Environ. 40, 962–976. doi: 10.1111/pce.12846, PMID: 27739594

[B34] Mittler R. Zandalinas S. I. Fichman Y. van Breusegem F. (2022). Reactive oxygen species signalling in plant stress responses. Nat. Rev. Mol. Cell Biol. 23, 663–679. doi: 10.1038/s41580-022-00499-2, PMID: 35760900

[B35] Ploughe L. W. Jacobs E. M. Frank G. S. Greenler S. M. Smith M. D. Dukes J. S. (2019). Community response to extreme drought (CRED): a framework for drought-induced shifts in plant-plant interactions. New Phytol. 222, 52–69. doi: 10.1111/nph.15595, PMID: 30449035

[B36] Qi M. Liu X. D. Li Y. B. Song H. Yin Z. T. Zhang F. . (2021). Photosynthetic resistance and resilience under drought, flooding and rewatering in maize plants. Photosynthesis Res. 148, 1–15. doi: 10.1007/s11120-021-00825-3, PMID: 33661466

[B37] Ren Y. Gao G. L. Ding G. D. Zhang Y. Zhao P. S. (2023). Temporal approach to identifying ectomycorrhizal community associated with Mongolian pine in a desert environment, northern China. Microbiol. Spectr. 11, e02026–e02023. doi: 10.1128/spectrum.02026-23, PMID: 37707453 PMC10580992

[B38] Ruehr N. K. Grote R. Mayr S. Arneth A. (2019). Beyond the extreme: recovery of carbon and water relations in woody plants following heat and drought stress. Tree Physiol. 39, 1285–1299. doi: 10.1093/treephys/tpz032, PMID: 30924906 PMC6703153

[B39] Sun C. S. Gao X. X. Chen X. Fu J. Q. Zhang Y. L. (2016). Metabolic and growth responses of maize to successive drought and re-watering cycles. Agr. Water Manage. 172, 62–73. doi: 10.1016/j.agwat.2016.04.016

[B40] Tedersoo L. Bahram M. Zobel M. (2020). How mycorrhizal associations drive plant population and community biology. Science 367, eaba1223. doi: 10.1126/science.aba1223, PMID: 32079744

[B41] Wang C. Chen J. Lee S. C. Xiong L. Su T. Lin Q. . (2025). Response and recovery times of vegetation productivity under drought stress: dominant factors and relationships. J. Hydrol. 655, 132945. doi: 10.1016/j.jhydrol.2025.132945

[B42] Wang Y. Q. Yang Z. F. Shi L. X. Yang R. Guo H. Zhang S. Q. . (2022). Transcriptome analysis of *Auricularia fibrillifera* fruit-body responses to drought stress and rehydration. BMC Genomics 23, 58. doi: 10.1186/s12864-021-08284-9, PMID: 35033026 PMC8760723

[B43] Xiao S. Liu L. T. Zhang Y. J. Sun H. K. Zhang K. Bai Z. U. . (2020). Fine root and root hair morphology of cotton under drought stress revealed with RhizoPot. J. Agron. Crop Sci. 206, 679–693. doi: 10.1111/jac.12429

[B44] Xu Z. Z. Zhou G. S. Shimizu H. (2010). Plant responses to drought and rewatering. Plant Signal. Behav. 5, 649–654. doi: 10.4161/psb.5.6.11398, PMID: 20404516 PMC3001553

[B45] Yin D. C. Song R. Q. Qi J. Y. Deng X. (2018). Ectomycorrhizal fungus enhances drought tolerance of *Pinus sylvestris* var. *mongolica* seedlings and improves soil condition. J. For. Res. 29, 1775–1788. doi: 10.1007/s11676-017-0583-4

[B46] Zhang H. Zhu J. Gong Z. Zhu J. K. (2022). Abiotic stress responses in plants. Nat. Rev. Genet. 23, 104–119. doi: 10.1038/s41576-021-00413-0, PMID: 34561623

[B47] Zhao P. S. Gao G. L. Ren Y. Ding G. D. Zhang Y. Wang J. Y. (2022). Intra-annual variation of root-associated fungi of *Pinus sylvestris* var. *mongolica*: The role of climate and implications for host phenology. App. Soil Ecol. 176, 104480. doi: 10.1016/j.apsoil.2022.104480

[B48] Zia R. Nawaz M. S. Siddique M. J. Hakim S. Imran A. (2021). Plant survival under drought stress: implications, adaptive responses, and integrated rhizosphere management strategy for stress mitigation. Microbiol. Res. 242, 126626. doi: 10.1016/j.micres.2020.126626, PMID: 33189069

