# Supplementary material for: Dynamic physiological response of Mongolian pine ectomycorrhizal seedlings to drought and re-watering
Source: Front Plant Sci. 2026 Feb 18;17:1744853. doi: 10.3389/fpls.2026.1744853 (PMC12957278; doi:10.3389/fpls.2026.1744853)
Supplement: Supplementary file 1 [file DataSheet1.docx]

**Supplementary Table 1** Two-factor ANOVA analysis of drought, re-watering and inoculation with EMF on leaf photosynthetic and fluorescence parameter of Mongolian pine seedlings.

|  | | Photosynthetic Parameter | | | |  | Fluorescence Parameter | | | |
| --- | --- | --- | --- | --- | --- | --- | --- | --- | --- | --- |
|  | | Tr | Pn | Ci | Gs |  | F_0_ | Fv/Fm | NPQ | qP |
| Drought-Rewatering | *F* | 54.793 | 60.330 | 28.070 | 56.483 |  | 31.890 | 60.099 | 119.187 | 658.296 |
|  | *P* | **<0.000** | **<0.000** | **<0.000** | **<0.000** |  | **<0.000** | **<0.000** | **<0.000** | **<0.000** |
| Contribution rate | % | 42.3 | 59.4 | 65.0 | 60.9 |  | 44.9 | 42.8 | 61.9 | 75.3 |
| Inoculation EMF | *F* | 44.428 | 13.769 | 10.753 | 8.566 |  | 10.099 | 65.767 | 59.261 | 228.313 |
|  | *P* | **<0.000** | **<0.000** | **<0.000** | **<0.000** |  | **<0.000** | **<0.000** | **<0.000** | **<0.000** |
| Contribution rate | % | 20.5 | 7.7 | 0.8 | 4.9 |  | 8.0 | 26.6 | 18.3 | 15.6 |
| Drought-Rewatering × Inoculation EMF | *F* | 0.238 | 0.098 | 4.234 | 0.247 |  | 0.120 | 0.302 | 1.078 | 2.627 |
|  | *P* | 0.999 | 1.000 | **<0.000** | 0.998 |  | 1.000 | 0.995 | 0.383 | **0.002** |
| Contribution rate | % | -1.1 | -2.7 | -3.4 | -2.6 |  | -3.8 | -1.1 | 0.3 | 0.6 |

**Supplementary Table 2** Two-factor ANOVA analysis of drought, re-watering and inoculation with EMF on water physiology parameter of Mongolian pine seedlings

|  |  | Leaf water | |  | WUE |  | Water potential | |
| --- | --- | --- | --- | --- | --- | --- | --- | --- |
|  |  | RWC | TD |  |  |  | ψ_l_ | ψr |
| Drought-Rewatering | *F* | 14.341 | 5.945 |  | 22.684 |  | 408.282 | 83.637 |
|  | *P* | **<0.000** | **<0.000** |  | **<0.000** |  | **<0.000** | **<0.000** |
| Contribution rate | % | 15.5 | 11.8 |  | 19.4 |  | 84.3 | 65.6 |
| Inoculation EMF | *F* | 71.655 | 9.747 |  | 29.427 |  | 67.661 | 7.938 |
|  | *P* | **<0.000** | **<0.000** |  | **<0.000** |  | **<0.000** | **<0.000** |
| Contribution rate | % | 49.3 | 12.5 |  | 24.2 |  | 8.3 | 3.5 |
| Drought-Rewatering × Inoculation EMF | *F* | 7.769 | 1.223 |  | 3.277 |  | 6.131 | 2.544 |
|  | *P* | **<0.000** | 0.262 |  | **<0.000** |  | **<0.000** | **0.02** |
| Contribution rate | % | 23.6 | 1.6 |  | 7.6 |  | 3.1 | 3.9 |

**Supplementary Table 3** Two-factor ANOVA analysis of drought, re-watering and inoculation with EMF on osmoregulation and antioxidant capacity of Mongolian pine seedlings

|  | | osmoregulatory substance | |  | cell membrane stability | |  | antioxidant enzyme activity | | |
| --- | --- | --- | --- | --- | --- | --- | --- | --- | --- | --- |
|  | | Pro | SS |  | REC | MDA |  | SOD | CAT | POD |
| Drought-Rewatering | *F* | 81.273 | 106.402 |  | 91.227 | 163.031 |  | 347.217 | 126.885 | 35.574 |
|  | *P* | **<0.000** | **<0.000** |  | **<0.000** | **<0.000** |  | **<0.000** | **<0.000** | **<0.000** |
| Contribution Rate | % | 37.2 | 76.2 |  | 70.6 | 71.8 |  | 84.4 | 46.49 | 64.92 |
| Inoculation EMF | *F* | 115.231 | 6.345 |  | 15.990 | 53.033 |  | 36.969 | 115.327 | 81.079 |
|  | *P* | **<0.000** | **<0.000** |  | **<0.000** | **<0.000** |  | **<0.000** | **<0.000** | **<0.000** |
| Contribution Rate | % | 31.8 | 2.3 |  | 7.0 | 13.8 |  | 5.3 | 25.33 | 1.37 |
| Drought-Rewatering × Inoculation EMF | *F* | 26.427 | 0.077 |  | 1.656 | 2.549 |  | 0.465 | 2.083 | 1.239 |
|  | *P* | **<0.000** | 1.000 |  | 0.068 | **0.002** |  | 0.954 | **0.014** | 0.251 |
| Contribution Rate | % | 35.3 | -2.0 |  | 1.5 | 1.5 |  | -0.4 | 1.2 | 1.91 |

**Supplementary Table 3** Correlations between the biochemical indicators and drought resistance, drought recovery, drought adaptability of Mongolian Pine seedlings under drought and re-watering conditions

| Item | Drought stress | | | Re-watering | | |
| --- | --- | --- | --- | --- | --- | --- |
|  | Drought resistance | Drought recovery | Drought adaptability | Drought resistance | Drought recovery | Drought adaptability |
| Tr | 0.372^**^ | 0.410^**^ | -0.120 | 0.448^**^ | 0.347^*^ | -0.042 |
| Pn | 0.416^**^ | 0.205 | -0.315^*^ | 0.395^**^ | 0.177 | -0.170 |
| Ci | 0.452^**^ | -0.047 | -0.344^*^ | 0.494^**^ | -0.182 | -0.050 |
| Gs | 0.428^**^ | 0.041 | -0.367^*^ | 0.498^**^ | -0.075 | -0.205 |
| F_0_ | 0.572^**^ | -0.039 | -0.085 | 0.615^**^ | 0.028 | 0.080 |
| Fv/Fm | 0.465^**^ | 0.446^**^ | 0.191 | 0.285^*^ | 0.614^**^ | 0.503^**^ |
| NPQ | 0.472^**^ | 0.396^**^ | 0.007 | 0.112 | 0.628^**^ | 0.066 |
| qP | 0.463^**^ | 0.461^**^ | 0.099 | 0.408^*^ | 0.474^**^ | 0.547^**^ |
| RWC | 0.354^*^ | 0.526^**^ | 0.382^*^ | 0.545^**^ | -0.107 | -0.078 |
| TD | 0.049 | 0.549^**^ | 0.429^**^ | -0.267 | 0.395^**^ | 0.331^*^ |
| WUE | 0.041 | -0.398^**^ | -0.334^*^ | -0.286^*^ | -0.417^**^ | -0.315^*^ |
| ψ_l_ | 0.524^**^ | 0.060 | -0.165 | 0.488^*^ | 0.073^**^ | 0.114 |
| ψ_r_ | 0.437^**^ | 0.114 | -0.146 | 0.509^**^ | -0.104 | 0.065 |
| Pro | -0.466^**^ | -0.411^**^ | -0.196 | -0.406^**^ | 0.111 | 0.409^**^ |
| SS | 0.311^*^ | 0.110 | 0.660^**^ | 0.208 | 0.104 | 0.741^**^ |
| REC | -0.531^**^ | -0.013 | 0.149 | -0.465^**^ | 0.138 | 0.227 |
| MDA | -0.391^**^ | -0.203 | 0.248 | -0.508^**^ | -0.072 | 0.087 |
| SOD | 0.386^**^ | 0.231 | 0.465^**^ | 0.319^*^ | 0.526^**^ | 0.282 |
| CAT | -0.087 | 0.734^**^ | 0.552^**^ | -0.005 | 0.739^**^ | 0.343^*^ |
| POD | 0.004 | 0.720^**^ | 0.665^**^ | -0.112 | 0.597^**^ | 0.557^*^ |

* indicates significant correlation at the α=0.05 level, ** indicates significant correlation at the α=0.01 level.

| 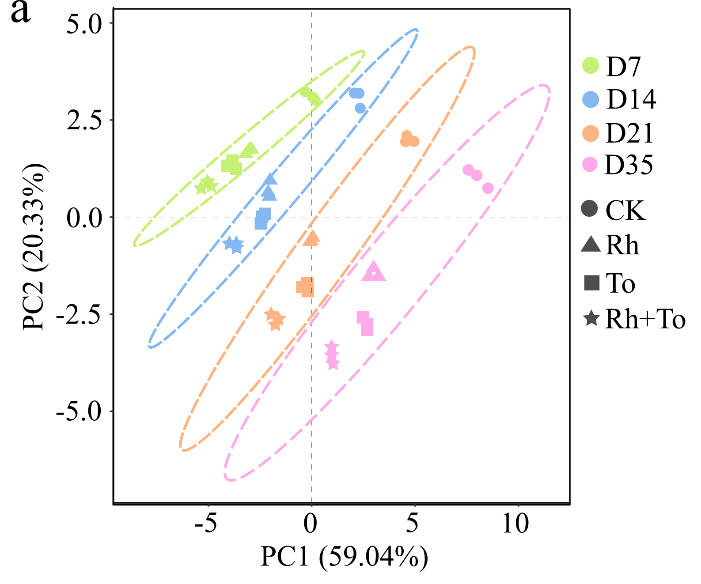 | 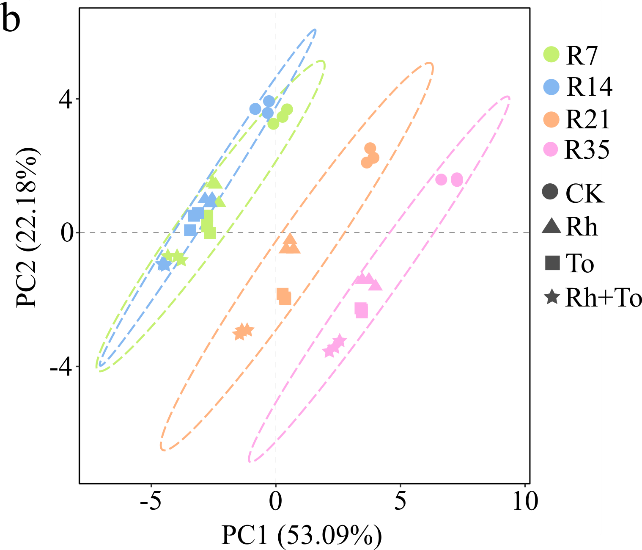 |
| --- | --- |
| **Figure S1.** PCA analysis of biochemical indicators of Mongolian pine seedlings under drought (a) and re-watering (b)  Note: different graphs represent different inoculation treatments, and different colored circles represent 95% confidence ellipses for different drought stress and re-watering groups. | |
